# Carcinosarcoma of the uterus prolapsing through the vagina

**DOI:** 10.1007/s00404-022-06530-3

**Published:** 2022-04-04

**Authors:** Felix Neis

**Affiliations:** Department of Obstetrics and Gynecology, University Hospital Tübingen, Calwerstrasse 7, 72076 Tübingen, Germany

**Keywords:** Carcinosarcoma, Delay of therapy, Tumor prolaps through vagina

**Fig. 1:**
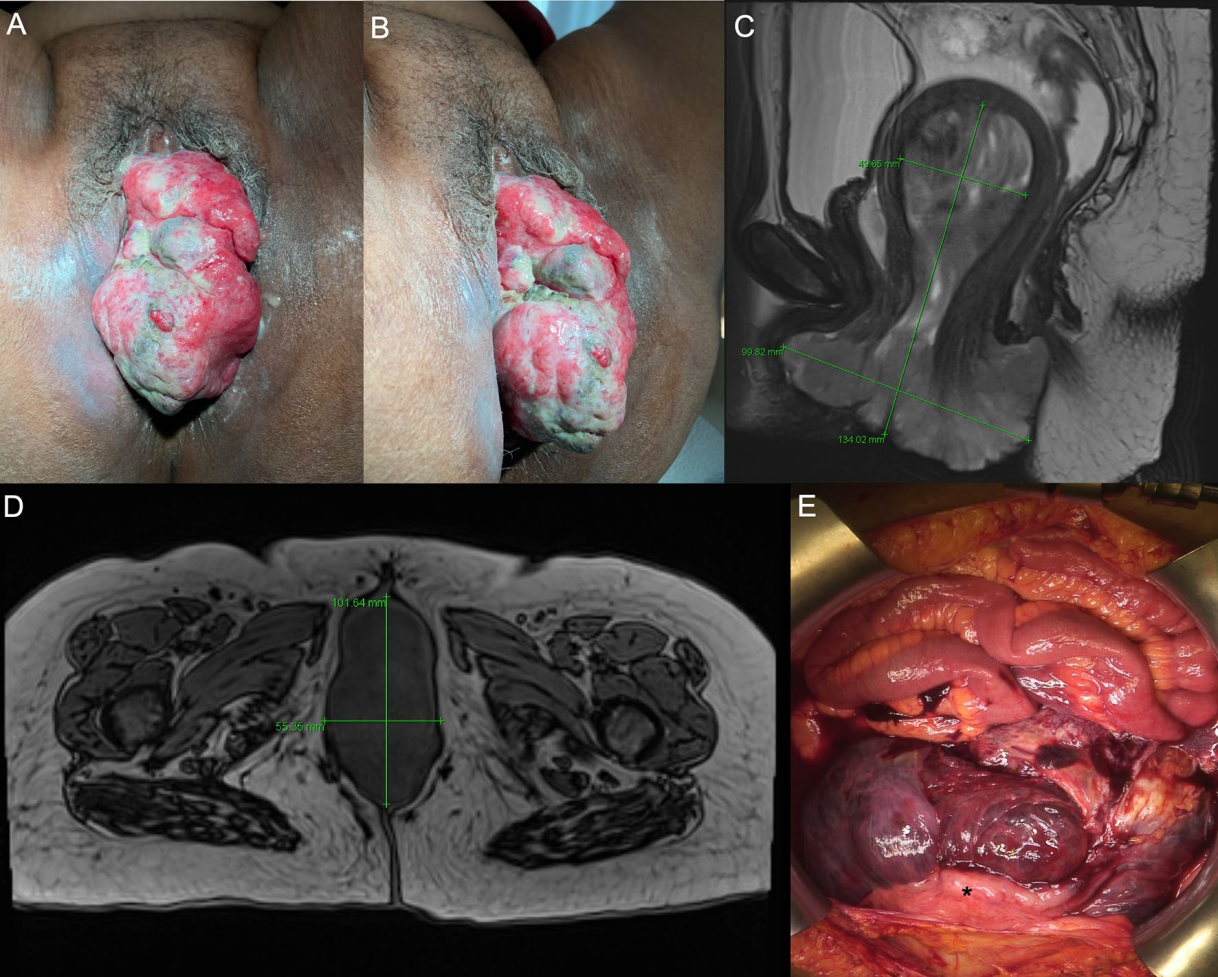
**A**, **B** External view of the tumor prolapsing out through the vagina. **A** View from the front. **B** View from the right side. **C**, **D** MRI scan of the pelvis, scale in mm. **C** Transverse view, T1 sequence. **D** Sagittal view, T2 sequence. **E** Intraoperative situs. 12 o’clock: cranial, 6 o’clock caudal. Lower abdomen filled with a gelatinous tumor. *Uterus

A 63-year-old Ethiopian female patient presented with a histologically confirmed high-grade, homologous carcinosarcoma of the uterus. 8 months after initial diagnosis without therapy, a 16 × 8 × 8 cm multilobulated exophytic tumor showed in front of the vulva (Fig. 1A, B).

MRI revealed an extensive proliferative formation growing from the uterine cavum, exophytically protruding transcervically in front of the vulva (Fig. 1C, D). The explorative laparotomy showed an inoperable situs with extensive, jelly-like tumor manifestations originating from the uterus which infiltrated the bladder, the rectosigmoid, and the small bowel (Fig. 1E). For bleeding control, the tumor was embolized via both internal iliac arteries. Since chemotherapy was refused by the patient, palliative therapy with best supportive care was initiated.

